# HIV-1 envelope, integrins and co-receptor use in mucosal transmission of HIV

**DOI:** 10.1186/1479-5876-9-S1-S2

**Published:** 2011-01-27

**Authors:** Claudia Cicala, James Arthos, Anthony S Fauci

**Affiliations:** 1 Laboratory of Immunoregulation National Institute of Allergy and Infectious Diseases, National Institutes of Health, Bethesda, MD 20892, USA

## Abstract

It is well established that HIV-1 infection typically involves an interaction between the viral envelope protein gp120/41 and the CD4 molecule followed by a second interaction with a chemokine receptor, usually CCR5 or CXCR4. In the early stages of an HIV-1 infection CCR5 using viruses (R5 viruses) predominate. In some viral subtypes there is a propensity to switch to CXCR4 usage (X4 viruses). The receptor switch occurs in ~ 40% of the infected individuals and is associated with faster disease progression. This holds for subtypes B and D, but occurs less frequently in subtypes A and C. There are several hypotheses to explain the preferential transmission of R5 viruses and the mechanisms that lead to switching of co-receptor usage; however, there is no definitive explanation for either. One important consideration regarding transmission is that signaling by R5 gp120 may facilitate transmission of R5 viruses by inducing a permissive environment for HIV replication. In the case of sexual transmission, infection by HIV requires the virus to breach the mucosal barrier to gain access to the immune cell targets that it infects; however, the immediate events that follow HIV exposure at genital mucosal sites are not well understood. Upon transmission, the HIV quasispecies that is replicating in an infected donor contracts through a “genetic bottleneck”, and often infection results from a single infectious event.  Many details surrounding this initial infection remain unresolved. In mucosal tissues, CD4^+^ T cells express high levels of CCR5, and a subset of these CD4^+^/CCR5^high^ cells express the integrin α_4_β_7_, the gut homing receptor. CD4^+^/CCR5^high^/ α4β7^high^ T cells are highly susceptible to infection by HIV-1 and are ideal targets for an efficient productive infection at the point of transmission. In this context we have demonstrated that the HIV-1 envelope protein gp120 binds to α_4_β_7_ on CD4^+^ T cells. On CD4^+^/CCR5^high^/ α4β7^high^ T cells, α_4_β_7_ is closely associated with CD4 and CCR5. Furthermore, α_4_β_7_ is ~3 times the size of CD4 on the cell surface, that makes it a prominent receptor for an efficient virus capture. gp120-α_4_β_7_ interactions mediate the activation of the adhesion-associated integrin LFA-1. LFA-1 facilitates the formation of virological synapses and cell-to-cell spread of HIV-1. gp120 binding to α_4_β_7_ is mediated by a tripeptide located in the V1/V2 domain of gp120. Of note, the V1/V2 domain of gp120 has been linked to variations in transmission fitness among viral isolates raising the intriguing possibility that gp120-α_4_β_7_ interactions may be linked to transmission fitness. Although many details remain unresolved, we hypothesize that gp120-α_4_β_7_ interactions play an important role in the very early events following sexual transmission of HIV and may have important implication in the design of vaccine strategies for the prevention of acquisition of HIV infection

## Introduction

Infection by HIV-1 causes a profound depletion of CD4^+^ T cells. This depletion eventually leads to the progression of HIV disease ultimately resulting in AIDS. The selective targeting of CD4^+^ T cells led to the seminal discovery that the CD4 receptor itself is the principal receptor targeted by HIV-1 [1,2]. To enter and infect a permissive cell, HIV-1 requires CD4 [1,2] and the chemokine coreceptors CCR5 or CXCR4 [3,4]. The chemokine receptor CCR5 is the predominant fusion cofactor for most transmitted HIV-1 strains [5].

## Gp120 exhibits a specific affinity for integrin α_4_β_7_

In the acute phase of infection after viral replication reaches high levels HIV-1 (in the case of humans) and SIV (in the case of non-human primates) destroy the majority of CD4^+^ T cells in draining lymphoid tissue, particularly gut-associated lymphoid tissue (GALT) [6-8]. This structural and functional damage is accompanied by nonspecific systemic immune activation and cell death [9,10] and many events surrounding HIV-1 replication in GALT have been elucidated [11-13]. Recently, we raised a new set of questions related to acute infection when we demonstrated that some HIV-1 isolates bind to and signal through integrin α_4_β_7_[14], the gut homing receptor.

α_4_β_7_ facilitates the migration of lymphocytes from gut inductive sites (Peyer’s patches and mesenteric lymph nodes) to the lamina propria [15]. These sites within GALT play central roles in the initial phases of infection following sexual transmission. α_4_β_7_^+^/CD4^+^ T cells have also been detected in genital mucosa [16-19], where CD4^+^ T cells are initially infected at the time of HIV transmission [6]. In this respect α_4_β_7_ provides a link between the earliest site of infection and gut inductive sites. Viruses isolated from acutely infected individuals replicate primarily in CD4^+^ T cells [20]. At the portal of infection in the female genital tract, i.e.vaginal, ecto- and endo-cervical tissues, CD4^+^ T-cells are dispersed within a few focal aggregates [21]. The early foci of infected CD4^+^ T cells are formed shortly after transmission and appear as small clusters of infected founder populations [22]. Within days, infected cells migrate from the genital mucosa to Peyer’s patches and mesenteric lymph nodes where high-level HIV replication occurs [6,23]. It is clear that α_4_β_7_ is functionally linked to each of the sites involved in the earliest phases of acute infection.

Unlike the HIV-1 entry receptors (CD4 and CCR5), α_4_β_7_ is not required for viral replication *in vitro*. Yet, the explicit linkage between α_4_β_7_, Peyer’s patches, mesenteric lymph nodes, lamina propria and the earliest phases of acute infection, suggests that gp120-α_4_β_7_ interactions play an important role at an early point in HIV infection *in vivo*. Supporting this proposition, α_4_β_7_ reactivity is conserved across gp120s from the four major HIV-1 subtypes. gp120 binding to α_4_β_7_ is mediated by a conserved tripeptide in the V2-loop. This tripeptide mimics a related tripeptide encoded by the natural ligands of α_4_β_7_[24]. This structural mimicry, along with the high degree of conservation across subtypes, implies that binding to α_4_β_7_ confers a replication advantage to HIV-1. A detailed understanding of the events surrounding transmission provide important clues as to how HIV-1 is utilizing α_4_β_7_ during the earliest days of infection in a new host.

Numerous barriers reduce the efficiency of mucosal transmission.  Studies of couples discordant for HIV infection have demonstrated that heterosexual transmission is very inefficient since multiple exposures typically precede successful transmission [25,26]. Given that sexual transmission is inefficient, and our determination that α_4_β_7_ CD4^+^ T cells are highly susceptible to productive infection [16] it is reasonable to assume that the ability of a virus to bind to α_4_β_7_ may be particularly relevant at an early stage of infection. Because different isolates of HIV-1 vary greatly in their α_4_β_7_ reactivity it is possible that viral isolates that exhibit optimal α_4_β_7_ reactivity are able to establish infection more efficiently. Specifically, the affinity of gp120 for α_4_β_7_ provides a mechanism for HIV-1 to target a subset of CD4^+^ T cells that are highly susceptible to infection. Such an activity may be particularly critical during transmission.

## α_4_β_7_ and CCR5 are coexpressed on a CD4^+^ T-cell subset that is highly susceptible to infection, which may favor the transmission of R5 viruses

**Figure 1:**
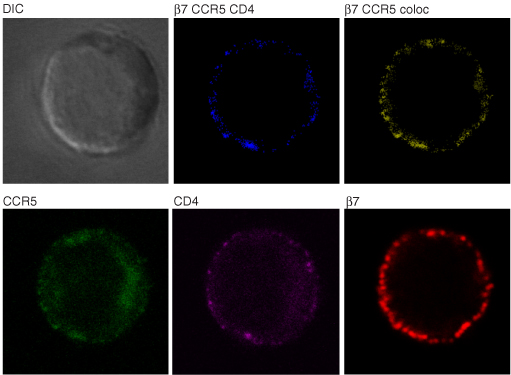
**On gut α_4_β_7_^high^ CD4^+^ T cells α_4_β_7_ colocalizes with CD4 and CCR5. **Freshly isolated gut cells obtained from biopsies taken from healthy donors were stained with the α_4_β_7_ mAb Act-1 (red), the CD4 mAb OKT4 (purple), and the CCR5 mAb 2D7 (green) and viewed under a confocal microscope. Unstained and individual stains of a representative cell are presented along with digitally defined regions of colocalization between α_4_β_7_ and CD4 (yellow) and α_4_β_7_, CD4 and CCR5 (blue). This cell is representative of greater that 60 cells analyzed from four donors.

**Figure 2:**
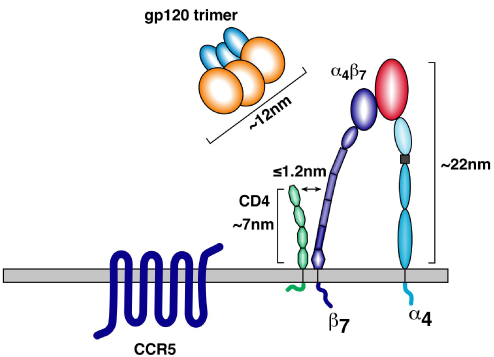
A schematic depicting approximate sizes of α_4_β_7_, CD4, and a gp160 trimer.

α_4_β_7_ is upregulated on activated CD4^+^ T cells localized within mucosal tissues that are highly relevant to HIV-1 pathogenesis: Peyer’s patches, mesenteric lymph nodes, lamina propria, and genital mucosa [15,16,19,27,28]. These cells also express high levels of CCR5, and therefore represent an ideal target population for productive infection. On these cells α_4_β_7_ is closely associated with both CD4, the HIV-1 entry receptor [16] (Fig. 1) and CCR5, the predominant fusion coreceptor. Of note, these cells express relatively low levels of CXCR4 [16]. Transmission of R5 viruses is strongly favored over X4 viruses; however, the underlying basis for the selection of R5 viruses is unknown. The marked coexpression of CCR5 and α_4_β_7_ along with the close physical association of these two surface markers with CD4, on cells that are highly susceptible to productive infection, may provide at least in part an explanation for the strong bias toward R5 virus transmission across mucosal surfaces. It is important to note that, despite the high level expression of CCR5 on this cellular subset, CCR5 is effectively hidden from HIV-1 before engaging the CD4 receptor.  In contrast, α_4_β_7_ is a prominent receptor (~3 times the size of CD4) (Fig 2) that gp120 can engage independently of CD4 [14,16]. Unlike CD4, which is expressed uniformly on both resting and activated CD4^+^ T cells, α_4_β_7_ is expressed at high levels primarily on activated cells. In this manner α_4_β_7_ provides a structural mechanism for HIV-1 to target activated cells that express high levels of CCR5.  Of note, analysis of subtype C gp120s derived from early transmitted isolates indicates that these gp120s require high levels of CCR5 and CD4 [29]. Thus, the structural prominence of α_4_β_7_ and its CD4-independent engagement of gp120, combined with its selective expression on metabolically activated [16] CCR5^high^ CD4^+^ T cells provides a rational basis for the HIV-1 envelope to have evolved a specific affinity for α_4_β_7_.

Of note, dendritic cells may play an important role in the selective transmission of R5 viral isolates via DC-T cells interaction as demonstrated *in vitro *[30,31]. At genital mucosa it is possible that antigen-specific T cells responses that involve DCs interacting with α_4_β_7_^+^ CD4^+^ T cells promote infection of those CD4^+^ T cells. In this regard it is noteworthy that the antigen-specific response to certain sexually transmitted diseases (STDs) involves α_4_β_7_^+^ CD4^+^ T cells [17,19]. Because the great majority of α_4_β_7_^+^ CD4^+^ T cells in the genital mucosa express high levels of CCR5 it is reasonable to envision that, in the contest of a STD-induced immune response, DC-T cell interactions in the genital mucosa may promote infection of CD4^+^ T-cells by R5 viruses.

## Signaling mediated by gp120

α_4_β_7_ is closely associated with CD4, the HIV-1 entry receptor, on CD4^+^ T cells in GALT. The interaction between gp120 and α_4_β_7_ triggers a signal, that is not yet fully defined [14,16]; however, it has been reported that the gp120-mediated signal transduction in several cellular subsets impacts viral replication. In this regard, a number of reports conclude that HIV-1 gp120 mediates signals that facilitate viral replication [32-36].  In this regard, HIV-1 gp120 is a unique ligand that can mediate signals in a near simultaneous manner through CD4, a chemokine receptor and α_4_β_7_. The first gp120-mediated signal to be reported involved a protein tyrosine kinase. In response to gp120 treatment, CD4^+^ T-cells rapidly phosphorylate p56lck, which then dissociates from the cytoplasmic domain of CD4 [37]. The identification of chemokine receptors as HIV coreceptors opened up new questions regarding the role of chemokine receptor signaling in viral infection and pathogenesis [3,4,38]. gp120 was shown to trigger rapid calcium fluxes by engaging CCR5 [39]. There is some evidence suggesting that the differential capacity of genetically distinct gp120s to signal correlates with their capacity to facilitate replication [32]. HIV-1 gp120 induces phosphorylation of several proteins, many involved in cytoskeleton rearrangement, including Pyk2 [40] and FAK [41]. Binding of gp120 to both CCR5 and CXCR4, activates several intracellular signaling cascades, mimicking the natural ligands of the chemokine receptors. HIV-1 gp120 has also been shown to trigger signaling in resting cells. In resting cells gp120 mediates the nuclear translocation of the transcription factor NFAT that can enhance viral transcription by binding to NFAT recognition sites on the HIV long terminal repeat (LTR) [33]. gp120 can mediate chemotaxis, actin cytoskeleton rearrangement [42] and the activation of an actin depolymerization factor, cofilin, in resting cells [43]. The density of cell surface CCR5 determines post-entry efficiency of replication of an R5 virus [44] and in unstimulated primary T cells, CCR5 signaling supports HIV-1 infection [45]. Moreover, gp120-CCR5 signaling can induce a distinct gene expression profile in primary cells and a signaling cascade, associated with cellular activation, that favors viral replication in non-proliferating target cells [33,34]. As noted above, R5 viruses dominate the early stages of infection, largely infecting activated memory CD4^+^ T cells in the draining lymphoid tissue, particularly the GALT [6,46-48]. Both activated and “ostensibly resting” CD4^+^ T cells are involved in the early stages of infection in the GALT [6]. The capacity of gp120 to trigger signals that promote viral replication in both activated and resting cells, may facilitate infection. This activity may be particularly important during mucosal transmission. Studies of transmission in an SIV macaque model [6,47,49] indicate that the first cells infected are not fully activated. It is in these cells that gp120 signals may provide the necessary metabolic stimulus to achieve productive infection.  Although our knowledge of gp120-α_4_β_7_ signaling is incomplete, we can speculate that it is in this setting that gp120-α_4_β_7_ signal transduction may play an important role and may be a major factor in the transmission of HIV at the mucosal surface.

## Virological synapse

HTLV-1 was the first retrovirus shown to spread through structures termed virological synapses (VS) [50]. It is now recognized that HIV-1 also spreads efficiently between T-lymphocytes across VS [51-56]. A VS is closely related in structure to an immunological synapse (IS) that is formed between an antigen presenting cells and T cells, an event that precedes T cell activation. Both IS and VS formation involves the dynamic movement of receptors at the membrane interface, leading to the formation of a signalosome [57]. Both the IS and the VS involve polarized structures that depend on adhesion molecules and cytoskeleton remodeling. VS allow efficient transfer of viral proteins from an infected cell to an uninfected target cell [50,52,55,56]. The formation of VS serves to promote a fast and potent signaling cascade of phosphorylation events, Ca^++^ flux and the activation of several genes. There occurs a significant active remodeling of the actin cytoskeleton that facilitates lateral mobility of receptors on the membrane. Moreover, local actin remodeling stabilizes cell-cell contacts and promotes efficient signal transmission [58-60]. Analogous mechanisms are likely at work in productive HIV-1 spread between CD4^+^ T-cells [61]. The similarity between the HIV-1 envelope-mediated signaling events and the signaling involved in the IS formation is remarkable [57]. However, despite similarities to IS, gp120-induced signaling mediates distinct membrane-proximal events that cause only a partial activation of canonical TCR signals [62,63]. It is therefore clear that the synapse mediated by HIV-1 uses some, but not all of the components of a classical IS.

**Figure 3:**
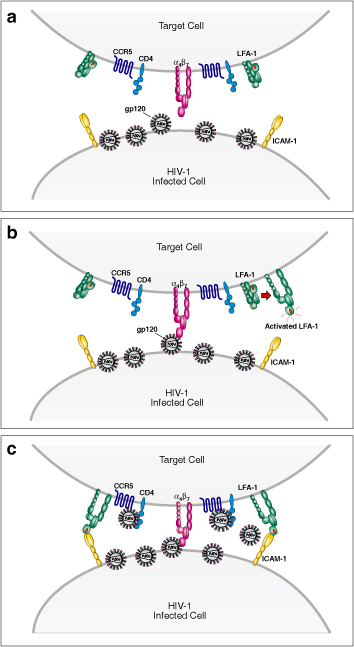
**A schematic depicting the formation of a Virological Synapse (VS) upon engagement of α_4_β_7_ by HIV-1 envelope. **An HIV-1 infected cell encounters a highly susceptible target cell expressing high levels of α_4_β_7_ (panel a). HIV-1 envelope on the surface of the infected cell binds to α_4_β_7_ on the target cell and activates the downstream integrin LFA-1 (panel b). LFA-1 binds to its ligand ICAM-1 (panel c) and stabilizes a VS.

Several reports are in agreement that HIV-1 transmission in T-lymphocytes cultures occurs predominantly through cell-cell spread with an estimated efficiency 100-1000 times greater than cell free virus replication [51,55,64-66]. The formation of an HIV-1 VS is facilitated by the interaction of envelope with CD4 and the chemokine coreceptor [52,53]. Integral to HIV-1 VS are adhesion molecules including LFA-1 and its ligand ICAM. Of note, gp120-α_4_β_7_ interactions mediate a rapid activation of LFA-1 [14] (Fig. 3a, 3b, 3c). It is important to emphasize that cell-to-cell spread of HIV through VS is far more efficient than cell free infection, and likely to be an important means of viral replication *in vivo*.

## HIV-1 envelope structure

The gp120 envelope glycoprotein of the HIV-1 promotes virus entry by sequentially binding CD4 and chemokine receptors on the target cell. Primary clinical HIV-1 isolates require an initial interaction with CD4 that induces conformation changes which allow gp120 to bind the CCR5 chemokine receptor in an efficient way. HIV-1 gp120 is composed of 5 variable regions (V1 to V5), and 5 constant regions (C1 to C5) that are generally similar among different viral isolates. Among the variable regions, disulfide bridges form the V1/V2 loop domain that joins the V1 and V2 loops. The other variable regions (V3, V4, V5) form independent loops in gp120 [67]. Advances in our understanding of the structure and function of the variable and constant regions of gp120 may provide insight into the design of a subunit immunogen for an HIV vaccine [68].

The envelope is in a non-covalently associated trimer of gp120/gp41 heterodimers. After gp120 binds CD4 it undergoes an extensive conformational change that rearranges both the gp120 core and the variable loops. Among the better-defined conformational changes are those involving the V3 loop [67]. Upon CD4 binding, the V3 loop is exposed and engages the coreceptor (CCR5 or CXCR4), which then mediates membrane fusion. A region of gp120 termed the ”bridging sheet” also interacts directly with the amino terminus of the coreceptor. The HIV envelope structure exhibits a plastic character and the variable loops contribute to this flexibility [69,70]. The V1/V2 loop is generally exposed on the envelope and is highly immunogenic. Of note, it is one of the first targets of the early immune response, and anti-V1/V2 Abs often have strong neutralizing activity against autologous virus at a relatively early time point after infection [71-74].

Several crystal structures of the core fragment of gp120 have been solved [75-78], many of which are missing the more flexible elements such as V1/V2, V3 loop, N- and C-terminal peptides as well as all of the post-translationally added carbohydrates [75-78]. HIV-1 gp120 structures were originally determined in complex with the D1D2 fragment of CD4 [77] or CD4 mimics [79], whereas one SIV gp120 structure was solved in an unliganded state [75]. Modeling on the crystal structure of a gp120 core suggests that the V3 loop is partially occluded by the V1/V2 loop when gp120 is in an unliganded state [75]. Despite dramatic antigenic differences between primary and laboratory-adapted HIV-1, key structural features of their gp120 cores are remarkably similar.

HIV-1 neutralization resistance is promoted by quaternary interactions involving the major variable loops. There is increasing evidence that sequence variation in the V1/V2 loop, and to a lesser extent V4 and V5 loops mediates neutralization resistance [69,74,80-82].  This variation occurs without serious detriment to the envelope core structure [76]. It has been reported that sequence variation in the V1/V2 loop plays an important role in viral tropism and in the regulation of cell entry [83-86]. Both the structure and function of the V1/V2 loop domain is poorly understood. However, in HIV subtypes A and C there exists a potential link between V1/V2 loop glycosylation, length and virus transmission [73,74,80,82,87-89]. In some studies, sequence analysis of gp120s derived from transmission pairs suggests that early-replicating isolates encode a “transmission signature” that includes sequences within the V1/V2 loop. This holds for both vertical and sexual transmission [74,88,90]. The first biological function assigned to the V1/V2 loop is the ability to bind to the integrin α_4_β_7_on the surface of CD4+ T cells [14].

## V1/V2 loop and transmission

Upon transmission across mucosal surfaces, the HIV quasispecies replicating in an infected donor contracts through a “genetic bottleneck” in the recipient, and often infection results from a single infectious event [89]. In some studies this genetic bottleneck does not appear to be random, but rather involves a bias toward the transmission of viruses encoding common genotypic characteristics. In HIV subtypes A and C the genetic signature of these founder/early transmitting viruses has been mapped primarily to the V1/V2 loop, with additional sites in C3 and V4. The two features that are most consistently overrepresented in early transmitting viruses are compact/shorter V1/V2s with reduced numbers of N-linked glycosylation sites relative to the quasispecies replicating in the plasma of donors at the time of transmission [73,74,80,82,87-89]. It has been suggested that these features increase, in some unknown manner, the transmission fitness of founder viruses [82,90]. Over time these genotypic features are diluted as the quasispecies heterogeneity increases [20,74,81,88,90].  Many questions surrounding the transmission bottleneck remain unanswered. In particular, there is no clear explanation as to how viruses encoding shortened V1/V2s with fewer N-linked glycosylation sites possess increased transmission fitness. In addition it is unclear why such features are found in some cohorts, but not others. Finally, it is unclear whether the genotypic signature of early transmitting viruses is similar in all viral subtypes and whether these patterns hold in all modes of transmission.

It is noteworthy that the genotypic signature of founder/early transmitting viruses maps to the V1/V2 loop that also includes a key binding site for α_4_β_7_[14].

To the extent that α_4_β_7_-reactivity facilitates infection, optimal α_4_β_7_ binding may, under certain conditions, facilitate transmission. Transmission is a critical point at which the virion must productively infect susceptible target cells. Once this occurs, active replication foci are established. This scenario has been termed the “broadcast” model of transmission [6,49]. The productive infection of susceptible target cells is a critical step. At this point in transmission, failure to infect highly susceptible cells will likely lead to an abortive transmission. The very first step in infection is the engagement of cell surface receptors by virion associated envelope spikes. If contact occurs with a susceptible target cell, a cascade of signals is triggered and a productive infection is initiated. Although many details remain unknown, it is reasonable to speculate that virions (or envelope spikes on infected cells) are likely to first engage α_4_β_7_ and subsequently engage CD4. α_4_β_7_ resides in close proximity to CD4 on the surface of a T cell (Fig.1); however, it is structurally more prominent, rising three times higher of the surface of the cell than does CD4 (Fig. 2). The gp120 V2-loop, which mediates binding to α_4_β_7_, is positioned near the apex of an envelope spike [91]. In this way both α_4_β_7_ and its binding epitope on a virion spike are well positioned for the initial engagement between the virion and the target CD4^+^ T cell.

## HIV-1 Neutralizing antibody-responses target the gp120 V1V2 loop

In general the neutralizing antibody responses to HIV-1 *in vivo* are of limited breadth [92]. This is particularly true for the V1/V2 loop. Autologous neutralizing antibodies against V1/V2 are frequently detected within months following infection [69,71,73,74,80,81]. Such responses, in many cases are potent and exert pressure on the envelope of HIV. However, the structural flexibility of V1/V2 facilitates rapid neutralization escape. A limited number of human broadly neutralizing mAbs have been isolated [93]. All of these well-characterized broadly neutralizing mAbs target the HIV-1 envelope. An extensive analysis of these antibodies, including their crystal structures has provided important information. All of these mAbs exhibit unusual structural characteristics.  Recently, two broadly neutralizing mAbs, PG9 and PG16, specific to the V1/V2 loop have been identified [94]. The identification of the two mAbs, that target conserved residues embedded within the variable loops suggest that variable loops can be considered as potential targets for a gp120-based vaccine immunogen [94,95].

## Conclusion

In natural infection the HIV-1 envelope protein is the primary target of neutralizing antibodies [67,96]. For this reason HIV-1 gp120 has been a central focus of efforts to develop subunit vaccine immunogens that can elicit neutralizing antibodies. The receptor binding epitopes on gp120 are conserved, and antibodies directed against these sites neutralize HIV-1, making receptor binding sites attractive targets in the context of an immunogen. These efforts have proven to be difficult because the viral envelope uses multiple mechanisms to evade and escape neutralizing responses. The envelope protein is hyper-variable in sequence, both within a patient and across each of the major clades. In addition the envelope encodes a shifting pattern of glycosylation. Finally, the flexibility of the variable loops results in conformational masking of conserved epitopes. In particular, the CD4 binding site on gp120, which is structurally conserved, is masked by glycans and variable loops. Thus, efforts to develop an immunogen capable of eliciting broadly cross-reactive Abs against the CD4 binding site have thus far been unsuccessful.

Unlike the CD4 binding site that the HIV envelope “hides” from antibodies, the α_4_β_7_ binding site in the gp120 V1/V2-loop is likely to be accessible, and immunogenic [70]. Moreover anti V1/V2-loop Ab responses are able to neutralize autologous virus [70,73,74,97]. The challenge presented by V1/V2 is that the predominant neutralizing responses against this domain are type-specific. V1/V2 readily escapes these responses through genetic drift and selection. What remains to be determined is whether conserved neutralizing epitopes can be identified and used to develop an effective immunogen. The potential utilization of α_4_β_7_ on susceptible CD4+ T cell targets by early-transmitting gp120s may hold important clues in this regard.

In conclusion, the specific affinity of gp120 for α_4_β_7_ may play a critical role in the infection of CD4^+^ T cells in mucosal tissues. α_4_β_7_ is not required for infection, but appears to increase the efficiency of infection. Moreover, α_4_β_7_ is closely associated with CD4 on a subset of CD4^+^ T cells that express high levels of CCR5 and are highly susceptible to infection. Signals delivered by gp120 through all three receptors have the capacity to promote productive infection. These observations suggest that gp120 engagement of α_4_β_7_ facilitates infection of CD4^+^ T cells in the earliest stages of transmission.  In future studies it will be important to determine if antibody responses directed against the V1/V2 region of gp120 can contribute to a protective immune response. In this regard further understanding the structural and functional interactions between V1/V2 and α_4_β_7_ may provide important information for the design of gp120 subunit vaccines.

## Competing interests

The author declare that they have no competing interests.

## References

[B1] Dalgleish AG The CD4 (T4) antigen is an essential component of the receptor for the AIDS retrovirus. Nature 1984 312 5996 763 7 10.1038/312763a0 6096719

[B2] Klatzmann D T-lymphocyte T4 molecule behaves as the receptor for human retrovirus LAV. Nature 1984 312 5996 767 8 10.1038/312767a0 6083454

[B3] Alkhatib G CC CKR5: a RANTES, MIP-1alpha, MIP-1beta receptor as a fusion cofactor for macrophage-tropic HIV-1. Science 1996 272 5270 1955 8 10.1126/science.272.5270.1955 8658171

[B4] Feng Y HIV-1 entry cofactor: functional cDNA cloning of a seven-transmembrane, G protein-coupled receptor. Science 1996 272 5263 872 7 10.1126/science.272.5263.872 8629022

[B5] Lederman MM Biology of CCR5 and its role in HIV infection and treatment. JAMA 2006 296 7 815 26 10.1001/jama.296.7.815 16905787

[B6] Haase AT Perils at mucosal front lines for HIV and SIV and their hosts. Nat Rev Immunol 2005 5 10 783 92 16200081 10.1038/nri1706

[B7] Picker LJ Immunopathogenesis of acute AIDS virus infection. Curr Opin Immunol 2006 18 4 399 405 10.1016/j.coi.2006.05.001 16753288

[B8] Veazey RS Gastrointestinal tract as a major site of CD4+ T cell depletion and viral replication in SIV infection. Science 1998 280 5362 427 31 10.1126/science.280.5362.427 9545219

[B9] Brenchley JM Douek DC The mucosal barrier and immune activation in HIV pathogenesis. Curr Opin HIV AIDS 2008 3 3 356 61 10.1097/COH.0b013e3282f9ae9c 19372990 PMC2789390

[B10] Brenchley JM Price DA Douek DC HIV disease: fallout from a mucosal catastrophe? Nat Immunol 2006 7 3 235 9 10.1038/ni1316 16482171

[B11] Brenchley JM Douek DC HIV infection and the gastrointestinal immune system. Mucosal Immunol 2008 1 1 23 30 10.1038/mi.2007.1 19079157 PMC2777614

[B12] McMichael AJ The immune response during acute HIV-1 infection: clues for vaccine development. Nat Rev Immunol 10 1 11 23 10.1038/nri2674 20010788 PMC3119211

[B13] Mehandru S Mechanisms of gastrointestinal CD4+ T-cell depletion during acute and early human immunodeficiency virus type 1 infection. J Virol 2007 81 2 599 612 10.1128/JVI.01739-06 17065209 PMC1797467

[B14] Arthos J HIV-1 envelope protein binds to and signals through integrin alpha(4)beta(7), the gut mucosal homing receptor for peripheral T cells. Nat Immunol 2008 18264102 10.1038/ni1566

[B15] Johansson-Lindbom B Agace WW Generation of gut-homing T cells and their localization to the small intestinal mucosa. Immunol Rev 2007 215 226 42 10.1111/j.1600-065X.2006.00482.x 17291292

[B16] Cicala C The integrin {alpha}4{beta}7 forms a complex with cell-surface CD4 and defines a T-cell subset that is highly susceptible to infection by HIV-1. Proc Natl Acad Sci U S A 2009 19933330 10.1073/pnas.0911796106 PMC2780317

[B17] Kelly KA Rank RG Identification of homing receptors that mediate the recruitment of CD4 T cells to the genital tract following intravaginal infection with Chlamydia trachomatis. Infect Immun 1997 65 12 5198 208 9393816 10.1128/iai.65.12.5198-5208.1997 PMC175749

[B18] Kelly KA Differential regulation of CD4 lymphocyte recruitment between the upper and lower regions of the genital tract during Chlamydia trachomatis infection. Infect Immun 2000 68 3 1519 28 10.1128/IAI.68.3.1519-1528.2000 10678969 PMC97310

[B19] Kelly KA The Combination of the Gastrointestinal Integrin (alpha4beta7) and Selectin Ligand Enhances T-Cell Migration to the Reproductive Tract During Infection with Chlamydia trachomatis. Am J Reprod Immunol 2009 10.1111/j.1600-0897.2009.00705.x PMC2891913 19392980

[B20] Salazar-Gonzalez JF Genetic identity, biological phenotype, and evolutionary pathways of transmitted/founder viruses in acute and early HIV-1 infection. J Exp Med 2009 206 6 1273 89 10.1084/jem.20090378 19487424 PMC2715054

[B21] Pudney J Quayle AJ Anderson DJ Immunological microenvironments in the human vagina and cervix: mediators of cellular immunity are concentrated in the cervical transformation zone. Biol Reprod 2005 73 6 1253 63 10.1095/biolreprod.105.043133 16093359

[B22] Schacker T Productive infection of T cells in lymphoid tissues during primary and early human immunodeficiency virus infection. J Infect Dis 2001 183 4 555 62 10.1086/318524 11170980

[B23] Mehandru S Primary HIV-1 infection is associated with preferential depletion of CD4+ T lymphocytes from effector sites in the gastrointestinal tract. J Exp Med 2004 200 6 761 70 10.1084/jem.20041196 15365095 PMC2211967

[B24] Zeller Y Mechtersheimer S Altevogt P Critical amino acid residues of the alpha4 subunit for alpha4beta7 integrin function. J Cell Biochem 2001 83 2 304 19 10.1002/jcb.1197 11573247

[B25] Gray RH Probability of HIV-1 transmission per coital act in monogamous, heterosexual, HIV-1-discordant couples in Rakai, Uganda. Lancet 2001 357 9263 1149 53 10.1016/S0140-6736(00)04331-2 11323041

[B26] Wawer MJ Rates of HIV-1 transmission per coital act, by stage of HIV-1 infection, in Rakai, Uganda. J Infect Dis 2005 191 9 1403 9 10.1086/429411 15809897

[B27] Berlin C alpha 4 integrins mediate lymphocyte attachment and rolling under physiologic flow. Cell 1995 80 3 413 22 10.1016/0092-8674(95)90491-3 7532110

[B28] von Andrian UH Mackay CR T-cell function and migration. Two sides of the same coin. N Engl J Med 2000 343 14 1020 34 10.1056/NEJM200010053431407 11018170

[B29] Alexander M Donor and recipient envs from heterosexual human immunodeficiency virus subtype C transmission pairs require high receptor levels for entry. J Virol 84 8 4100 4 10.1128/JVI.02068-09 20147398 PMC2849512

[B30] David SA Selective transmission of R5-tropic HIV type 1 from dendritic cells to resting CD4+ T cells. AIDS Res Hum Retroviruses 2001 17 1 59 68 10.1089/088922201750056799 11177384

[B31] Yamamoto T Selective transmission of R5 HIV-1 over X4 HIV-1 at the dendritic cell-T cell infectious synapse is determined by the T cell activation state. PLoS Pathog 2009 5 1 e1000279 10.1371/journal.ppat.1000279 19180188 PMC2627922

[B32] Arthos J CCR5 signal transduction in macrophages by human immunodeficiency virus and simian immunodeficiency virus envelopes. J Virol 2000 74 14 6418 24 10.1128/JVI.74.14.6418-6424.2000 10864653 PMC112149

[B33] Cicala C HIV-1 gp120 induces NFAT nuclear translocation in resting CD4+ T-cells. Virology 2006 345 1 105 14 10.1016/j.virol.2005.09.052 16260021

[B34] Cicala C HIV envelope induces a cascade of cell signals in non-proliferating target cells that favor virus replication. Proc Natl Acad Sci U S A 2002 99 14 9380 5 10.1073/pnas.142287999 12089333 PMC123149

[B35] Kinter AL HIV envelope induces virus expression from resting CD4+ T cells isolated from HIV- infected individuals in the absence of markers of cellular activation or apoptosis. J Immunol 2003 170 5 2449 2455 12594269 10.4049/jimmunol.170.5.2449

[B36] Yu D The HIV envelope but not VSV glycoprotein is capable of mediating HIV latent infection of resting CD4 T cells. PLoS Pathog 2009 5 10 e1000633 10.1371/journal.ppat.1000633 19851458 PMC2760144

[B37] Juszczak RJ Effect of human immunodeficiency virus gp120 glycoprotein on the association of the protein tyrosine kinase p56lck with CD4 in human T lymphocytes. J Biol Chem 1991 266 17 11176 83 2040625

[B38] Berger EA Murphy PM Farber JM Chemokine receptors as HIV-1 coreceptors: roles in viral entry, tropism, and disease. Annu Rev Immunol 1999 17 657 700 10.1146/annurev.immunol.17.1.657 10358771

[B39] Weissman D Macrophage-tropic HIV and SIV envelope proteins induce a signal through the CCR5 chemokine receptor. Nature 1997 389 6654 981 5 10.1038/40173 9353123

[B40] Davis CB Signal transduction due to HIV-1 envelope interactions with chemokine receptors CXCR4 or CCR5. J Exp Med 1997 186 10 1793 8 10.1084/jem.186.10.1793 9362541 PMC2199136

[B41] Cicala C Induction of phosphorylation and intracellular association of CC chemokine receptor 5 and focal adhesion kinase in primary human CD4+ T cells by macrophage-tropic HIV envelope. J Immunol 1999 163 1 420 6 10384144

[B42] Balabanian K CXCR4-tropic HIV-1 envelope glycoprotein functions as a viral chemokine in unstimulated primary CD4+ T lymphocytes. J Immunol 2004 173 12 7150 60 15585836 10.4049/jimmunol.173.12.7150

[B43] Yoder A HIV envelope-CXCR4 signaling activates cofilin to overcome cortical actin restriction in resting CD4 T cells. Cell 2008 134 5 782 92 10.1016/j.cell.2008.06.036 18775311 PMC2559857

[B44] Lin YL The efficiency of R5 HIV-1 infection is determined by CD4 T-cell surface CCR5 density through G alpha i-protein signalling. AIDS 2006 20 10 1369 77 10.1097/01.aids.0000233570.51899.e2 16791011

[B45] Lin YL G-protein signaling triggered by R5 human immunodeficiency virus type 1 increases virus replication efficiency in primary T lymphocytes. J Virol 2005 79 12 7938 41 10.1128/JVI.79.12.7938-7941.2005 15919952 PMC1143625

[B46] Brenchley JM CD4+ T cell depletion during all stages of HIV disease occurs predominantly in the gastrointestinal tract. J Exp Med 2004 200 6 749 59 10.1084/jem.20040874 15365096 PMC2211962

[B47] Li Q Peak SIV replication in resting memory CD4+ T cells depletes gut lamina propria CD4+ T cells. Nature 2005 434 7037 1148 52 15793562 10.1038/nature03513

[B48] Mehandra S The gastrointestinal tract is critical to the pathogenesis of acute HIV-1 infection. J Allergy Clin Immunol 2005 116 2 419 422 10.1016/j.jaci.2005.05.040 16083799

[B49] Haase AT Targeting early infection to prevent HIV-1 mucosal transmission. Nature 464 7286 217 23 10.1038/nature08757 20220840

[B50] Igakura T Spread of HTLV-I between lymphocytes by virus-induced polarization of the cytoskeleton. Science 2003 299 5613 1713 6 10.1126/science.1080115 12589003

[B51] Chen P Predominant mode of human immunodeficiency virus transfer between T cells is mediated by sustained Env-dependent neutralization-resistant virological synapses. J Virol 2007 81 22 12582 95 10.1128/JVI.00381-07 17728240 PMC2169007

[B52] Jolly C HIV-1 cell to cell transfer across an Env-induced, actin-dependent synapse. J Exp Med 2004 199 2 283 93 10.1084/jem.20030648 14734528 PMC2211771

[B53] Piguet V Sattentau Q Dangerous liaisons at the virological synapse. J Clin Invest 2004 114 5 605 10 15343375 10.1172/JCI22812 PMC514595

[B54] Sherer NM Retroviruses can establish filopodial bridges for efficient cell-to-cell transmission. Nat Cell Biol 2007 9 3 310 5 10.1038/ncb1544 17293854 PMC2628976

[B55] Sol-Foulon N ZAP-70 kinase regulates HIV cell-to-cell spread and virological synapse formation. EMBO J 2007 26 2 516 26 10.1038/sj.emboj.7601509 17215865 PMC1783460

[B56] Sowinski S Membrane nanotubes physically connect T cells over long distances presenting a novel route for HIV-1 transmission. Nat Cell Biol 2008 10 2 211 9 10.1038/ncb1682 18193035

[B57] Haller C Fackler OT HIV-1 at the immunological and T-lymphocytic virological synapse. Biol Chem 2008 389 10 1253 60 10.1515/BC.2008.143 18713012

[B58] Billadeau DD Nolz JC Gomez TS Regulation of T-cell activation by the cytoskeleton. Nat Rev Immunol 2007 7 2 131 43 10.1038/nri2021 17259969

[B59] Sancho D Regulation of microtubule-organizing center orientation and actomyosin cytoskeleton rearrangement during immune interactions. Immunol Rev 2002 189 84 97 10.1034/j.1600-065X.2002.18908.x 12445267

[B60] Sechi AS Wehland J Interplay between TCR signalling and actin cytoskeleton dynamics. Trends Immunol 2004 25 5 257 65 10.1016/j.it.2004.03.003 15099566

[B61] Jolly C Sattentau QJ Retroviral spread by induction of virological synapses. Traffic 2004 5 9 643 50 10.1111/j.1600-0854.2004.00209.x 15296489

[B62] Vasiliver-Shamis G Human immunodeficiency virus type 1 envelope gp120-induced partial T-cell receptor signaling creates an F-actin-depleted zone in the virological synapse. J Virol 2009 83 21 11341 55 10.1128/JVI.01440-09 19710135 PMC2772796

[B63] Vasiliver-Shamis G Human immunodeficiency virus type 1 envelope gp120 induces a stop signal and virological synapse formation in noninfected CD4+ T cells. J Virol 2008 82 19 9445 57 10.1128/JVI.00835-08 18632854 PMC2546991

[B64] Blanco J High level of coreceptor-independent HIV transfer induced by contacts between primary CD4 T cells. J Biol Chem 2004 279 49 51305 14 10.1074/jbc.M408547200 15371410

[B65] Dimitrov DS Quantitation of human immunodeficiency virus type 1 infection kinetics. J Virol 1993 67 4 2182 90 8445728 10.1128/jvi.67.4.2182-2190.1993 PMC240333

[B66] Sourisseau M Inefficient human immunodeficiency virus replication in mobile lymphocytes. J Virol 2007 81 2 1000 12 10.1128/JVI.01629-06 17079292 PMC1797449

[B67] Hoxie JA Toward an Antibody-Based HIV Vaccine. Annu Rev Med 2009 10.1146/annurev.med.60.042507.164323 19824826

[B68] Poignard P gp120: Biologic aspects of structural features. Annu Rev Immunol 2001 19 253 74 10.1146/annurev.immunol.19.1.253 11244037

[B69] Lynch RM Appreciating HIV type 1 diversity: subtype differences in Env. AIDS Res Hum Retroviruses 2009 25 3 237 48 10.1089/aid.2008.0219 19327047 PMC2853864

[B70] Pinter A Roles of HIV-1 Env variable regions in viral neutralization and vaccine development. Curr HIV Res 2007 5 6 542 53 10.2174/157016207782418470 18045110

[B71] Gray ES Neutralizing antibody responses in acute human immunodeficiency virus type 1 subtype C infection. J Virol 2007 81 12 6187 96 10.1128/JVI.00239-07 17409164 PMC1900112

[B72] Moore PL The c3-v4 region is a major target of autologous neutralizing antibodies in human immunodeficiency virus type 1 subtype C infection. J Virol 2008 82 4 1860 1869 10.1128/JVI.02187-07 18057243 PMC2258729

[B73] Rong R Role of V1V2 and other human immunodeficiency virus type 1 envelope domains in resistance to autologous neutralization during clade C infection. J Virol 2007 81 3 1350 9 10.1128/JVI.01839-06 17079307 PMC1797511

[B74] Rong R Escape from autologous neutralizing antibodies in acute/early subtype C HIV-1 infection requires multiple pathways. PLoS Pathog 2009 5 9 e1000594 10.1371/journal.ppat.1000594 19763269 PMC2741593

[B75] Chen B Structure of an unliganded simian immunodeficiency virus gp120 core. Nature 2005 433 7028 834 41 10.1038/nature03327 15729334

[B76] Kwong PD Structure of an HIV gp120 envelope glycoprotein in complex with the CD4 receptor and a neutralizing human antibody. Nature 1998 393 6686 648 59 10.1038/31405 9641677 PMC5629912

[B77] Ryu SE Crystal structure of an HIV-binding recombinant fragment of human CD4. Nature 1990 348 6300 419 26 10.1038/348419a0 2247146 PMC5638305

[B78] Zhu P Distribution and three-dimensional structure of AIDS virus envelope spikes. Nature 2006 441 7095 847 52 10.1038/nature04817 16728975

[B79] Huang CC Scorpion-toxin mimics of CD4 in complex with human immunodeficiency virus gp120 crystal structures, molecular mimicry, and neutralization breadth. Structure 2005 13 5 755 68 10.1016/j.str.2005.03.006 15893666

[B80] Moore PL Gray ES Morris L Specificity of the autologous neutralizing antibody response. Curr Opin HIV AIDS 2009 4 5 358 63 10.1097/COH.0b013e32832ea7e8 20048698 PMC3004050

[B81] Moore PL Limited neutralizing antibody specificities drive neutralization escape in early HIV-1 subtype C infection. PLoS Pathog 2009 5 9 e1000598 10.1371/journal.ppat.1000598 19763271 PMC2742164

[B82] Sagar M Human immunodeficiency virus type 1 V1-V2 envelope loop sequences expand and add glycosylation sites over the course of infection, and these modifications affect antibody neutralization sensitivity. J Virol 2006 80 19 9586 98 10.1128/JVI.00141-06 16973562 PMC1617272

[B83] Cheng-Mayer C Selection for neutralization resistance of the simian/human immunodeficiency virus SHIVSF33A variant in vivo by virtue of sequence changes in the extracellular envelope glycoprotein that modify N-linked glycosylation. J Virol 1999 73 7 5294 300 10364275 10.1128/jvi.73.7.5294-5300.1999 PMC112584

[B84] Stamatatos L Wiskerchen M Cheng-Mayer C Effect of major deletions in the V1 and V2 loops of a macrophage-tropic HIV type 1 isolate on viral envelope structure, cell entry, and replication. AIDS Res Hum Retroviruses 1998 14 13 1129 39 10.1089/aid.1998.14.1129 9737584

[B85] Sullivan N Effect of amino acid changes in the V1/V2 region of the human immunodeficiency virus type 1 gp120 glycoprotein on subunit association, syncytium formation, and recognition by a neutralizing antibody. J Virol 1993 67 6 3674 9 8497077 10.1128/jvi.67.6.3674-3679.1993 PMC237724

[B86] Wyatt R Involvement of the V1/V2 variable loop structure in the exposure of human immunodeficiency virus type 1 gp120 epitopes induced by receptor binding. J Virol 1995 69 9 5723 33 7543586 10.1128/jvi.69.9.5723-5733.1995 PMC189432

[B87] Chohan B Selection for human immunodeficiency virus type 1 envelope glycosylation variants with shorter V1-V2 loop sequences occurs during transmission of certain genetic subtypes and may impact viral RNA levels. J Virol 2005 79 10 6528 31 10.1128/JVI.79.10.6528-6531.2005 15858037 PMC1091724

[B88] Derdeyn CA Hunter E Viral characteristics of transmitted HIV. Curr Opin HIV AIDS 2008 3 1 16 21 10.1097/COH.0b013e3282f2982c 19372939

[B89] Keele BF Derdeyn CA Genetic and antigenic features of the transmitted virus. Curr Opin HIV AIDS 2009 4 5 352 7 10.1097/COH.0b013e32832d9fef 20048697

[B90] Wu X Neutralization escape variants of human immunodeficiency virus type 1 are transmitted from mother to infant. J Virol 2006 80 2 835 44 10.1128/JVI.80.2.835-844.2006 16378985 PMC1346878

[B91] Liu J Molecular architecture of native HIV-1 gp120 trimers. Nature 2008 455 7209 109 13 10.1038/nature07159 18668044 PMC2610422

[B92] Wyatt R Sodroski J The HIV-1 envelope glycoproteins: fusogens, antigens, and immunogens. Science 1998 280 5371 1884 8 10.1126/science.280.5371.1884 9632381

[B93] Burton DR HIV vaccine design and the neutralizing antibody problem. Nat Immunol 2004 5 3 233 6 10.1038/ni0304-233 14985706

[B94] Walker LM Broad and potent neutralizing antibodies from an African donor reveal a new HIV-1 vaccine target. Science 2009 326 5950 285 9 10.1126/science.1178746 19729618 PMC3335270

[B95] Zolla-Pazner S Cardozo T Structure-function relationships of HIV-1 envelope sequence-variable regions refocus vaccine design. Nat Rev Immunol 2010 10 7 527 35 10.1038/nri2801 20577269 PMC3167078

[B96] Pantophlet R Burton DR GP120: target for neutralizing HIV-1 antibodies. Annu Rev Immunol 2006 24 739 69 10.1146/annurev.immunol.24.021605.090557 16551265

[B97] Gray ES Antibody specificities associated with neutralization breadth in plasma from human immunodeficiency virus type 1 subtype C-infected blood donors. J Virol 2009 83 17 8925 37 10.1128/JVI.00758-09 19553335 PMC2738176

